# The Regulatory Role of Non-coding RNAs on Programmed Cell Death Four in Inflammation and Cancer

**DOI:** 10.3389/fonc.2019.00919

**Published:** 2019-09-18

**Authors:** Mengxiang Zhao, Nisha Zhu, Fengyao Hao, Yuxian Song, Zhiyong Wang, Yanhong Ni, Liang Ding

**Affiliations:** ^1^Central Laboratory Nanjing Stomatological Hospital, Medical School of Nanjing University, Nanjing, China; ^2^Department of Oral and Maxillofacial Surgery, Nanjing Stomatological Hospital, Nanjing, China

**Keywords:** PDCD4, miRNA, lncRNA, cancer, inflammation

## Abstract

Programmed cell death 4 (PDCD4) is a tumor suppressor gene implicated in many cellular functions, including transcription, translation, apoptosis, and the modulation of different signal transduction pathways. The downstream mechanisms of PDCD4 have been well-discussed, but its upstream regulators have not been systematically summarized. Noncoding RNAs (ncRNAs) are gene transcripts with no protein-coding potential but play a pivotal role in the regulation of the pathogenesis of solid tumors, cardiac injury, and inflamed tissue. In recent studies, many ncRNAs, especially microRNAs (miRNAs) and long noncoding RNAs (lncRNAs), were found to interact with PDCD4 to manipulate its expression through transcriptional regulation and function as oncogenes or tumor suppressors. For example, miR-21, as a classic oncogene, was identified as the key regulator of PDCD4 by targeting its 3′-untranslated region (UTR) to promote tumor proliferation, migration, and invasion in colon, breast, and bladder carcinoma. Therefore, we reviewed the recently emerging pleiotropic regulation of PDCD4 by ncRNAs in cancer and inflammatory disorders and aimed to shed light on the mechanisms of associated diseases, which could be conducive to the development of novel treatment strategies for PDCD4-induced diseases.

## Introduction

### The Function and Structure of PDCD4

The main functions of Programmed cell death 4 (PDCD4)(NCBI GeneID: 27250) are reflected in the following two aspects. First, it acts as a suppressor in tumor progression; second, it is an inflammatory factor that participates in inflammation (1–3). An alteration in PDCD4 expression is pivotal to the pathogenesis of cancer and inflammation diseases. The expression of PDCD4 is downregulated in many kinds of human cancers, such as breast carcinoma, hepatocellular carcinoma, oral carcinoma, and ovarian cancer (4–7). The overexpression of PDCD4 induces apoptosis or cell cycle arrest, inhibits the invasion, proliferation and migration of cancer cells, and increases the sensitivity of cancer cells to antineoplastic drugs (8–11). In addition, knockdown of PDCD4 expression by an siRNA or shRNA stimulates invasion and migration in nasopharyngeal and lung cancer cells (12, 13). In brief, aberrant PDCD4 expression levels are associated with the progression of multiple diseases. Understanding the regulatory mechanisms of PDCD4 expression and targeting the homeostasis of PDCD4 is beneficial for related treatment. Thus, therapeutic strategies based on PDCD4 manipulation are promising treatments for cancer or inflammatory disorders.

**Figure 1:**
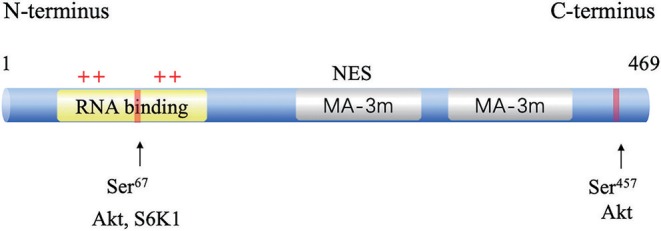
Structure and functional sites of PDCD4. The two conserved MA-3 domains are in green, the phosphorylation sites are shown in red, and the RNA-binding site in blue.

The human gene *PDCD4* is located at human chromosome 10q24 (14). The PDCD4 protein was identified independently from different species, including humans, mice, and chickens. The deduced amino acid sequences are highly conserved among these species (15). *PDCD4* encodes a 469-amino acid peptide composed of two conserved alpha helical MA3 domains (amino acids 164–275 and 329–440). These two domains are also present in eukaryotic translation initiation factors, eIF4G I, and eIF4G II (16). A yeast two-hybrid assay (16), a mammalian two-hybrid assay and analyses of the PDCD4-eIF4A cocrystal structure revealed that PDCD4 interacts with eIF4A by its MA-3 domains, limits ribosomal recombination and protein synthesis and inhibits malignant behaviors (17). eIF4A1(NCBI GeneID: 1973) is an RNA helicase that catalyzes the unwinding of the secondary structure at the 5′-untranslated region (UTR) of mRNAs. PDCD4 binds to two molecules of eIF4A through its MA3 domains to inhibit translation initiation by preventing eIF4G from binding to eIF4A (18). The PDCD4 protein contains two nuclear export signals (NESs), suggesting that the protein might be able to shuttle between the nucleus and cytoplasm (19). The phosphorylation of PDCD4 by Akt and S6K1 at Ser67 and Ser457 causes the nuclear translocation of PDCD4, contributing to its ubiquitination via an E3 ligase β-transducin repeat-containing protein (β-TRCP) and subsequent proteasome-dependent degradation (20, 21). It has also been reported that PDCD4 binds to RNA through its two positively charged amino acid clusters, RBM1 and RBM2, at the N-terminal domain [(22); Figure 1].

## Downstream Signals of PDCD4

**Figure 2:**
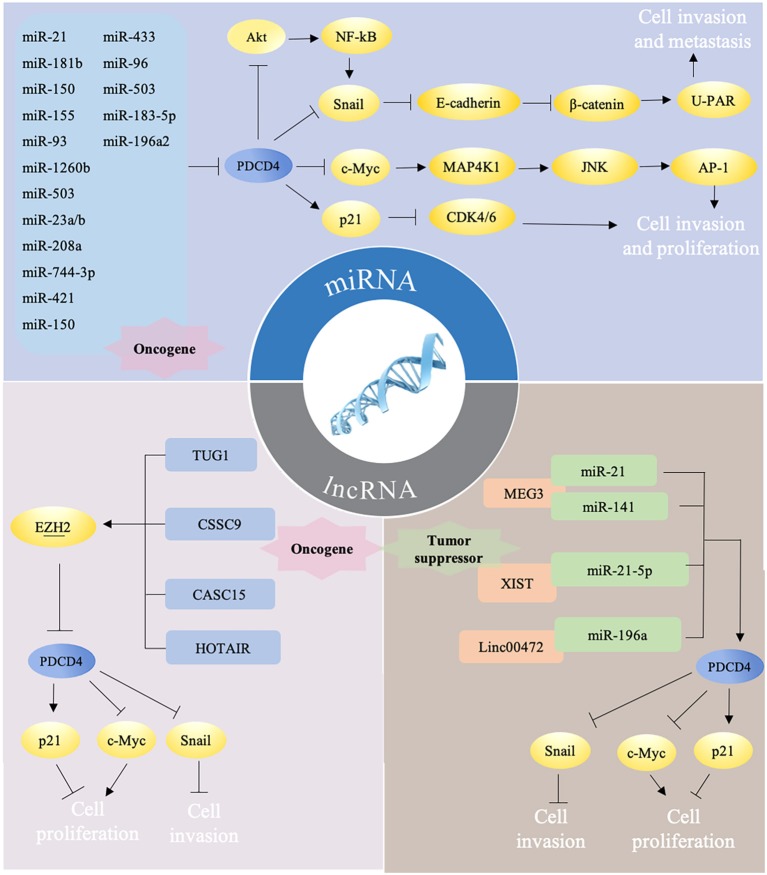
Different ncRNAs regulate PDCD4 in mutiple ways. (i) miRNAs target PDCD4 directly to promote tumor progression via several pathways. Briefly, PDCD4 reduces the expression of Snail directly or by NF-κB/Akt pathway and leads to up-regulation of E-cadherin, inhibition of β-catenin dependent transcription, and decrease of the expression of c-Myc and uPAR. Down-regulated c-Myc subsequently inhibits MAP4K1 expression, thereby inhibiting AP-1 transcription to impede proliferation, promotion, and invasion. PDCD4 could decrease CDK4/6 via the upregulation of p21, thus leading to reduced cell proliferation. (ii) LncRNAs could function as oncogene and downregulate PDCD4 expression by recruiting EZH2. (iii) LncRNAs could also function as tumor suprefessor to sponge miRNAs, which may counteract the effects of miRNAs on PDCD4.

PDCD4 are reported to participate into the control of several cellular signaling pathways. PDCD4 interacts and inhibits eIF4A and activator protein 1 (AP-1)-dependent transcription in a concentration-dependent manner through many transcription factors, including JNK, MAP4K1, c-Myc, E-cadherin, β-catenin, and Snail. The overexpression of PDCD4 in mouse epidermal JB6 cells inhibits both basal and 12-O-tetradecanoylphorbol-13-acetate (TPA)-induced AP-1 transactivation through the inhibition of c-Jun activation (23). In colon tumor cells, PDCD4 regulates the expression of the JNK upstream kinase MAP4K1 by c-Myc, resulting in the activation of JNK and c-Jun, to control the activation of AP-1. A mutation in the c-Myc binding site of the MAP4K1 promoter could reduce MAP4K1 promoter activity, and the downregulation of c-Myc can restore MAP4K1 expression and the activation of AP1 in *PDCD4*-knockdown colon tumor GEO and HT29 cells (24). In addition, *PDCD4* knockdown suppresses E-cadherin expression through elevated protein levels of Snail, causing the activation of β-catenin-dependent transcription and stimulating the expression of c-Myc and urokinase-type plasminogen activator (u-PAR) (25). u-PAR is a 55–60 kDa glycosylated receptor for the degradation of extracellular matrix components by binding to its ligand and allowing human osteosarcoma cells to penetrate the basal membrane during invasion (26). Snail is a transcriptional repressor that binds to E-boxes on the E-cadherin promoter for transcription inhibition (27). The regulation of Snail by PDCD4 was demonstrated through Akt, and the knockdown of Akt abolishes *PDCD4* knockdown-induced Snail expression in colon cancer (28). Akt can also activate NF-κB to upregulate Snail (29). PDCD4 was also found to inhibit carbonic anhydrase type II (CAII) expression in HEK-293 (human embryonic kidney) cells. CAII is an important substrate for the synthesis of amino acids, lipids, and pyrimidine for tumor growth (30). In addition, PDCD4 could decrease CDK4/6 (cyclin-dependent kinase 4/6) via the upregulation of p21^Waf1/Cip1^ and repress the CDK1 and cdc2 (cell division cycle 2) promoter in a neuroendocrine cell line, thus leading to reduced cell proliferation (31). By manipulating these pathways, PDCD4 ultimately inhibits cell survival, proliferation, and metastasis (Figure 2).

## ncRNAs as Upstream Regulators of PDCD4

The downstream mechanisms of PDCD4 have been well-discussed, but its upstream regulators have not been systematically summarized. In recent years, most studies have focused on the regulation of PDCD4 expression by noncoding RNAs (ncRNAs). In the following review, we will emphasize the regulation of PDCD4 expression by ncRNAs, which should provide a reference for upcoming clinical and laboratory studies on PDCD4 regulation.

Recent reports have revealed that ncRNAs contribute to the regulation of PDCD4 expression and function. ncRNAs are RNA molecules that cannot code proteins and were found to engage in the regulation of multiple cellular activities, including proliferation, differentiation, apoptosis, stress, and immune responses (32–36). ncRNAs consist of microRNAs (miRNAs), long noncoding RNAs (lncRNAs) and circular RNAs (circRNAs), which regulate specific gene expression through regulating transcriptional, posttranscriptional, and posttranslational processes. miRNA are 20–25 nucleotides in length, and mature miRNAs usually bind to the 3′-UTR of their target mRNAs through their seed sequences to cause the degradation of target mRNAs and to block translational protein synthesis. The seed sequence is usually located 2–7 nucleotides from the 5′-end of the miRNA and is complementary to a site in the 3′-UTR of its target mRNA (37). LncRNAs have more than 200 nucleotides in length with little protein-coding potential, which have been defined to regulate gene expression by transcriptional regulation, genetic imprinting, chromatin remodeling, posttranscriptional regulation, and translational regulation (38). In recent years, ncRNAs have been demonstrated to manipulate the availability of PDCD4 via different ways (Figure 2), including directly targeting the 3′-UTR of PDCD4 by a miRNA or epigenetic modification and miRNA sponging on PDCD4 by a lncRNA. ncRNAs, including miRNAs and lncRNAs, are key regulators of PDCD4 dosage, and delicately modulate PDCD4 expression (Table 1).

**Table 1:** The summary of ncRNA that regulate PDCD4 in different cancers and inflammatory disorders.

**Cancer**	**ncRNA**	**Functional responses and targets**	**References**
Laryngeal cancer	miR-503	miR-503 inhibits apoptosis by directly targeting PDCD4	(39)
	miR-744-3p	miR-744-3p regulates PDCD4 to reduce AKT and NF-κB activation as well as MMP-9 expression	(40)
Gastric cancer	miR-23a/b	miR-23a/b promotes tumor growth and suppress apoptosis by targeting PDCD4	(41)
	miR-208a-3p	miR-208a-3p suppresses cell apoptosis by targeting PDCD4	(42)
	miR-93	miR-93 functions as an oncomiR for the downregulation of PDCD4	(43)
	miR-196a2	miR-196a2 inhibits apoptosis by directly targeting PDCD4	(44)
Colorectal cancer	miR-1260b	miR-1260b inhibitor enhances the chemosensitivity 5-FU due to downregulation of PDCD4	(45)
	miR-181b	Activation of IL-6/STAT3 suppressed PDCD4 by upregulating miR-181b	(46)
Cervical cancer	miR-150	miR-150 functions as a tumor promoter in reducing chemosensitivity and promoting invasiveness via targeting PDCD4	(47)
Breast cancer	miR-421	PDCD4 is a direct target gene of miR-421	(10)
	miR-183-5p	Inhibition of miR-183-5p could repress the progression of breast cancer through restoring PDCD4 levels	(48)
Melanoma	CASC15	CASC15 acts as an oncogene by negatively regulating PDCD4 expression via recruiting EZH2 and subsequently increasing H3K27me3 level	(49)
	miR-150	Knockdown of miR-150 enhanced cell apoptosis via direct targeting of PDCD4	(50)
Osteosarcoma	miR-433	miR-433 suppresses the expression of PDCD4	(51)
Lung cancer	miR-155	miR-155 exerts an onco- genic role in NSCLC by directly targeting PDCD4	(52)
Liver cancer	miR-93	miR-93 dramatically promoted HCC invasion and metastasis by EMT via targeting PDCD4	(53)
Glioblastoma	miR-503	microRNA-503 increases proliferation of glioblastoma cells and inhibits apoptosis by directly targeting PDCD4	(54)
Osteosarcoma	XIST/miR-21-5p	lncRNA-XIST acts as a miRNA sponge, impedes miR-21-5p to maintain the expression of PDCD4	(55)
Colorectal cancer	Linc00472/miR-196a	Linc00472 suppressed proliferation and induced apoptosis through up-regulating PDCD4 by decoying miR-196a	(56)
Esophageal carcinoma	TUG1	TUG1 suppressed PDCD4 expression by recruiting EZH2 to the promoter region of PDCD4 and increasing H3K27me3 level in ESCC cells	(57)
	CASC9	lncRNA CASC9 functions as an oncogene by negatively regulating PDCD4 expression through recruiting EZH2 and subsequently altering H3K27me3 level	(58)
Glioma	HOTAIR	Suppression of PDCD4 mediated by HOTAIR inhibits glioma cell proliferation and invasion in a PRC2-dependent manner	(59)

## miRNA-Regulated PDCD4 Promotes Tumor Progression

Most recent studies of PDCD4 have focused on miRNAs, and now we will focus on regulatory mechanisms in tumors. More than 30 miRNAs were reported to be direct negative regulators of PDCD4, and many of them showed enhanced expression in tumors (40, 60). Furthermore, bioinformatics analyses predict that more than 80 miRNAs potentially target PDCD4, implying an essential role for miRNAs in regulating PDCD4 expression (61). These miRNAs downregulate PDCD4 levels and function by binding to the 3′-UTR of *PDCD4* mRNA. Among these miRNAs, studies conducted on miR-21(NCBI GeneID: 406991) are the most extensive. The human gene miR-21 was one of the first identified mammalian miRNAs and is located at chromosome 17q23.2 within the highly conserved gene encoding TMEM49 (62). Through early lineage tracing studies, miR-21 was found to be upregulated in various diseases, such as oropharyngeal cancer (63) and salivary adenoid cystic carcinoma (64). Asangani et al. performed a bioinformatic search and uncovered a potentially conserved site for miR-21 within the 3′-UTR of *PDCD4* mRNA and demonstrated that miR-21 inhibited PDCD4 levels to reduce the ability of invasion, intravasation and metastasis (65). miR-21 is associated with therapeutic outcome and poor survival in malignant cancer (66). For instance, miR-21 is overexpressed in salivary adenoid cystic carcinoma (SACC) cells, and the suppression of miR-21 with a miR-21 inhibitor in SACC cells could increase the activity of the PDCD4 promoter and the expression of PDCD4 protein, suppress p-STAT3 protein expression, through further feedback, reduce miR-21 expression, and finally lead to the inhibition of cell invasion and migration (64). Moreover, further recent studies confirmed the regulation of PDCD4 by miR-21 in colon, breast, and bladder carcinoma (67).

In colorectal cancer (CRC) cells, activated IL-6/STAT3 signals increased the expression of miR-181, which leaded to downregulating the expression of PDCD4 and promoting cell proliferation and metastasis and inhibit the apoptosis of CRC cells (46). In cervical cancer, overexpressed miR-150 can also promote the proliferation, migration and invasion of cervical cancer cells *in vitro* by directly targeting the expression of PDCD4 (47). miR-155(NCBI GeneID: 406947) decreases PDCD4 levels by binding to the 3′-UTR of PDCD4. PDCD4 is a functional target of miR-155 and regulates proliferation or invasion by targeting PDCD4 in non-small-cell lung cancer (52). In human hepatocellular carcinoma (HCC), the downregulation of PDCD4 by miR-93 (NCBI GeneID: 407050) promotes HCC cell migration and invasion via the epithelial-mesenchymal transition (EMT) pathway (68). In laryngeal squamous cell carcinoma, miR-744-3p (NCBI GeneID: 100126313) could activate the MMP-9 regulatory axis by provoking the signaling pathway controlled by NF-κB p65 by suppressing PDCD4 (40). The inhibition of miR-1260b induces a decrease in PDCD4 expression, as well as phosphorylated Akt (p-Akt) and phosphorylated extracellular signal-regulated kinase (p-ERK) (45). In glioblastoma, miRNA-503 (NCBI GeneID: 574506), induced by TGF-α1, inhibits apoptosis and increases the proliferation of glioblastoma cells by directly targeting PDCD4 (54). miR-421 (NCBI GeneID: 693122) regulates the proliferation, migration, invasion and apoptosis of breast cancer cells, including MCF-7 and MDA-MB-231 cells, by targeting PDCD4 (10). miR-433 (NCBI GeneID: 574034) is significantly overexpressed in osteosarcoma tissues and cell lines. The transfection of miR-433 mimics into osteosarcoma cell lines could decrease apoptosis by PDCD4. In contrast, the inhibition of miR-433 enhanced the apoptosis of tumor cells (51). Similarly, miR-23a/b (NCBI GeneID: 407010) (41), miR-208a-3p (NCBI GeneID: 406990) (42), miR-150 (NCBI GeneID: 406942)(47), miR-96 (NCBI GeneID: 407053) (69), miR-503 (NCBI GeneID: 574506) (39), miRNA-183-5p (NCBI GeneID: 406959) (48), and miR-196 (NCBI GeneID: 406972) also decrease protein levels by binding to the 3′-UTR of PDCD4 (44).

## miRNA-Mediated PDCD4 Downregulation Protects Against Inflammation

In addition to carcinogenesis, the regulation of PDCD4 by miRNAs also plays an important role in various inflammatory responses. Das et al. demonstrated that the miR-21/PDCD4 axis plays a key role in the process of turning on an anti-inflammatory phenotype in efferocytosis-the digestion and elimination of dead or dying cells by phagocytes. Elevated miR-21 in LPS-activated macrophages promotes efferocytosis and silences the target gene PDCD4, which in turn results in the elevated production of anti-inflammatory IL-10 and accounts for a net anti-inflammatory phenotype (70). The miR-21/PDCD4 axis also regulates mesenchymal stem cells (MSCs) to secrete stanniocalcin 1 (STC1) and other neuroprotective factors to inhibit retinal ganglion cell (RGC) apoptosis and microglial activation and promote RGC survival in a mouse model of acute glaucoma (71). The increased miR-21 expression level following spinal cord injury (SCI) may enhance neurite outgrowth to promote the repair of injured spinal cords by inhibiting the expression of PDCD4 (72). miR-16 targets and inhibits PDCD4 expression in atherosclerosis to suppress the activation of inflammatory macrophages through mitogen-activated protein kinase (MAPK) and NF-κB signaling, and suppresse the expression of proinflammatory factors, including interleukin (IL)-6 and tumor necrosis factor-α (TNF-α), whereas it enhanced the expression of the anti-inflammatory factor IL-10. Thus, the miR-16-PDCD4 axis suppresses the activation of inflammatory macrophages in atherosclerosis (73). In another study, the overexpression of miR-499 (NCBI GeneID: 574501) protected cardiomyocytes against LPS-induced apoptosis by inhibiting PDCD4. However, the experiment was performed in rats, and the experimental results may not be directly extrapolated to humans (74). Increased miRNA expression has also been reported in diseases caused by inflammation, including colitis and atherosclerosis. In these cases, triggering a regulatory response through miRNA would be beneficial. These findings suggest that the regulation of PDCD4 by miRNAs, in addition to its negative effects on tumors, can also play a positive role in other diseases.

## lncRNAs Act as Oncogenes to Regulate PDCD4 by Epigenetic Modification

LncRNAs have also been identified as critical regulators in a variety of cancer types [4], including epigenetic modification, transcriptional regulation, RNA decay, and miRNA sponging. Bioinformatic analysis revealed that the promoter region of PDCD4 was enriched in the repressive marker histone H3 lysine 27 trimethylation (H3 K27me3) and enhancer of zeste homolog 2 (EZH2) binding sites, demonstrating that PDCD4 expression is under the regulation of epigenetic modification (58). EZH2, an important catalytic subunit of polycomb repressive complex 2 (PRC2), is a histone methyltransferase that epigenetically represses target gene expression by promoting H3 K27me3 (57). Cancer susceptibility candidate 15 (CASC15) (NCBI GeneID: 401237), also named linc00340, is located on chromosome 6p22.3 and was initially identified as a highly active lncRNA (75). The expression of CASC15 is upregulated in melanoma, hepatocellular carcinoma and gastric cancer. It acts as an oncogene in cancer progression and phenotype switching. In melanoma, CASC15 may recruit EZH2, and EZH2 could subsequently directly bind to the promoter of PDCD4 in melanoma cells and inhibit PDCD4 expression (49). Wu et al. found that CASC9 (NCBI GeneID: 101805492) knockdown decreased the enrichment of EZH2 and H3K27me3 in the PDCD4 promoter region, which resulted in the upregulation of PDCD4 (58). Downregulation of the lncRNA HOTAIR (NCBI GeneID: 100124700) was demonstrated to activate the expression of PDCD4 at the transcriptional level in glioma stem cells by reducing the recruitment of downstream molecules, including EZH2 and LSD1 (76). The lncRNA taurine upregulated gene 1 (TUG1) (NCBI GeneID: 55000) was also demonstrated to suppress PDCD4 by recruiting EZH2 in esophageal squamous cell carcinoma (ESCC) through epigenetic modification (57, 59).

## lncRNAs Act as Tumor Suppressors to Regulate PDCD4 Through miRNA Sponging

The regulation of PDCD4 by lncRNAs is also promoted by another mechanism by which the dysregulated lncRNAs could sponge special miRNAs to suppress their target genes, such as PDCD4, through a competing endogenous RNA (ceRNA) mechanism. The ceRNA hypothesis accounts for the fact that specific RNAs are able to attenuate miRNA activity through sequestration and elevate miRNA target gene expression. The hypothesis potentially accounts for the function of a substantial proportion of the thousands of yet uncharacterized lncRNAs (77). LncRNAs block the effects of miRNAs via the competition for the seed sites of miRNAs with their target mRNAs. For instance, the lncRNA maternally expressed gene 3 (MEG3) (NCBI GeneID: 55384) is located at chromosome 14q32 and could enhance the sensitivity of colorectal cancer (CRC) cells to oxaliplatin via the upregulation of PDCD4 by sponging miR-141 and overcoming oxaliplatin resistance in CRC (78). In addition to its role in cancer, MEG3 also functions as a ceRNA for miR-21 to regulate PDCD4 expression in ischemic neuronal death followed by reperfusion (79). The lncRNA growth arrest-specific transcript 5 (GAS5) (NCBI GeneID: 60674) is downregulated in several kinds of cancers, including cervical cancer and breast cancer, and HCC tissues. The knockdown of GAS5 leads to the repression of cell viability. GAS5 as a ceRNA competes with PDCD4 to bind to miR-21, and the depletion or overexpression of GAS5 could lead to the downregulation or upregulation of PDCD4 levels in tumor cells. In HCCs, GAS5 acts as a tumor suppressor through the negative regulation of miR-21 and its target PDCD4 to suppress the migration and invasion of cancer cells (80). Similarly, GAS5 deficiency by siGAS5 also reduced miR-21 target protein PDCD4 expression in cervical cancer cells. The malignant behaviors of cervical cancer cells, manifested by cell migration and invasion, were enhanced by siGAS5 (81). Linc00472 (NCBI GeneID: 79940) is downregulated in CRC tissues and cells, and it acts as a tumor suppressor by upregulating PDCD4 by sponging miR-196a (56). miR-93-5p, a direct target of linc00472, directly targets PDCD4. The miR-93-5p/PDCD4 pathway mediated the suppressive role of linc00472 in HCC cells (82). The lncRNA DGCR5 and miR-320a regulate each other in a reciprocal manner, and DGCR5 could reverse the inhibition of PDCD4 by miR-320a, which is involved in the regulation of the pancreatic ductal adenocarcinoma cell phenotype (83). The expression of the lncRNA NBAT1 is downregulated in osteosarcoma tissues and cell lines. NBAT1 (NCBI GeneID: 729177) functions as a ceRNA against miR-21 to increase the expression of the miR-21 target gene PDCD4 and then suppresses osteosarcoma growth and metastasis *in vitro* and *in vivo* (84). Similarly, the lncRNA XIST inhibits cell growth and mobility by competitively binding to miR-21-5p for PDCD4 up-regulation in osteosarcoma (55).

These findings show that PDCD4 expression and activity are controlled by the network of ncRNAs, and the dysregulation of these ncRNAs can contribute to changes in PDCD4 function in various diseases.

## Targeting the ncRNA/PDCD4 Signaling Pathway: Therapeutic Applications

Since drug resistance commonly occurs in cancer patients, it is critical to develop alternative therapeutic strategies to resensitize resistant cancer cells and patient-derived models (PDX) (85, 86). Recently, published studies have demonstrated that the miRNA/PDCD4 axis could modulate chemosensitivity in resistant cancers. Treatment with a combination of drugs and miRNA inhibitors is a viable strategy for enhancing chemosensitivity though their synergistic effects. PDCD4 can downregulate the 5-fluorouracil (5-FU) resistance induced by miR-21 in pancreatic cancer cells and rescue the phenotypic characteristics disrupted by miR-21 (87). The role of miR-21/PDCD4 in drug resistance also concerns gemcitabine resistance in breast cancer, glioblastoma cancer, and pancreatic cancer.

In addition to affecting the resistance of cancer cells, there are also some drugs that can directly regulate the level of miRNA/PDCD4, thus serving as a potential therapeutic application. Treatment with isoalantolactone remarkably increased the expression of PDCD4 via the downregulation of miR-21, which exerts anticancer effects against esophageal squamous cell carcinoma (88). Quercetin is a kind of flavonoid that was reported to inhibit both acute and chronic Cr(VI)-induced miR-21 elevation and PDCD4 reduction in human bronchial epithelial cells. Besides, the Cr(VI)-induced binding of miR-21 to the 3′-UTR of PDCD4 was reduced by treatment with quercetin (89). It has been demonstrated that curcumin can inhibit tumor proliferation, invasion and metastasis by inhibiting the miR-21 transcription to stabilize the PDCD4 expression in CRC (90). In addition, the long intergenic noncoding RNA 152 (linc00152) was upregulated and promoted tumor progression and conferred oxaliplatin resistance in colon cancer by functioning as a ceRNA to release erb-b2 receptor tyrosine kinase 4 (ERBB4) by sponging miR-193a-3p (91).

The abovementioned evidence illustrates that targeting these transcription factors through small molecule drugs that regulate miRNA/PDCD4 expression may be effective in treating cancers.

## Conclusions

We here summarize the roles of miRNAs and lncRNAs in PDCD4 regulation. Recent studies have demonstrated that ncRNAs interact with PDCD4 at the transcriptional and posttranscriptional levels. Several miRNAs directly target PDCD4 to repress the PDCD4 protein level and function, which promotes tumorigenesis. Then, the miRNA/PDCD4 axis could exert a protective effect in inflammation by downregulating miRNA levels. lncRNAs have been reported to regulate PDCD4 in multiple ways. By epigenetic modification, a lncRNA could down-regulate PDCD4 expression by recruiting EZH2 to its promoter region and increasing the H3K27me3 enrichment of its promoter. In addition, as a miRNA sponge, lncRNAs also counteract the effects of miRNAs on their target mRNAs. The regulation of miRNAs by ceRNAs has added a new layer of complexity to PDCD4 regulation by miRNAs. However, there is also controversy about the ceRNA hypothesis. The essence of the doubt against the ceRNA hypothesis is that any change in the expression of an individual miRNA target would constitute only a small fraction of the target site abundance (77), implicating that physiological changes of one individual lncRNA might be insufficient to suppress miRNA activity.

## Discussion

The abovementioned studies deepen our understanding of the diverse roles of the ncRNA/PDCD4 pathway in inflammation and cancer. An urgent issue for clinicians is whether the ncRNA/PDCD4 pathway can be used as a potential target for therapeutic intervention; however, related clinical studies are lacking. Interestingly, miRNAs targeting PDCD4, including miR-21 and miR-23, have been tested in clinical trials via liposomes or other strategies for the treatment of inflammatory diseases and cancer (92). For example, treatment with anti-miR-21 oligonucleotides reduced breast cancer MCF-7 xenograft growth (93); therefore, PDCD4 might also be a new therapeutic target for cancer. However, based on the dual role of the ncRNA/PDCD4 pathway in tumor and inflammatory diseases, research on anticancer interventions must ensure that targeting ncRNA/PDCD4 would not render the side effects that induce an inflammatory response, such as endothelial inflammatory damage through the NF-κB/TNF-α signaling pathway (1). Since certain elevated levels of miRNAs and lncRNAs may also contribute to cancer promotion, more consideration should be taken in targeting of the ncRNA/PDCD4 pathway during the treatment of inflammatory diseases. Further dissection of ncRNAs in the PDCD4 pathway at the molecular and cellular levels will provide insights into the underlying mechanisms of PDCD4 in tumor suppression and devise novel avenues in drug development against cancer and other PDCD4-associated diseases.

### Author Contributions

MZ, NZ, FH, YS, ZW, YN, and LD collected the related paper and drafted the manuscript. MZ, NZ, ZW, YN, and LD participated in the design of the review and draft the manuscript. All authors read and approved the final manuscript.

### Conflict of Interest Statement

The authors declare that the research was conducted in the absence of any commercial or financial relationships that could be construed as a potential conflict of interest.
